# Pro-inflammatory cytokines and leukocyte integrins associated with chronic neuropathic pain in traumatic and inflammatory neuropathies: Initial observations and hypotheses

**DOI:** 10.3389/fimmu.2022.935306

**Published:** 2022-08-02

**Authors:** Chaoling Dong, Eroboghene E. Ubogu

**Affiliations:** Neuromuscular Immunopathology Research Laboratory, Division of Neuromuscular Disease, Department of Neurology, University of Alabama at Birmingham, Birmingham, Alabama, United States

**Keywords:** cytokines, leukocyte integrins, mouse models, neuroinflammation, nociception, peripheral neuropathy, sciatic nerve crush injury

## Abstract

Leukocyte infiltration and persistence within peripheral nerves have been implicated in chronic nociception pathogenesis in murine peripheral neuropathy models. Endoneurial cytokine and chemokine expression contribute to leukocyte infiltration and maintenance of a pro-inflammatory state that delays peripheral nerve recovery and promotes chronic pain behaviors in these mice. However, there has been a failure to translate murine model data into safe and effective treatments for chronic neuropathic pain in peripheral neuropathy patients, or develop reliable biomarkers that may help diagnose or determine treatment responses in affected patients. Initial work showed that persistent sciatic nerve CD11b+ CD45+ leukocyte infiltration was associated with disease severity in three mouse models of inflammatory and traumatic peripheral neuropathies, implying a direct contributing role in disease pathogenesis. In support of this, CD11b+ leukocytes were also seen in the sural nerve biopsies of chronic neuropathic pain patients with three different peripheral neuropathies. Systemic CD11b antagonism using a validated function-neutralizing monoclonal antibody effectively treated chronic nociception following unilateral sciatic nerve crush injury (a representative traumatic neuropathy model associated with axonal degeneration and increased blood-nerve barrier permeability) and does not cause drug addiction behaviors in adult mice. These data suggest that CD11b could be an effective molecular target for chronic neuropathic pain treatment in inflammatory and traumatic peripheral neuropathies. Despite known murine peripheral neuropathy model limitations, our initial work suggests that early expression of pro-inflammatory cytokines, such as tissue inhibitor of metalloproteinases-1 may predict subsequent chronic nociception development following unilateral sciatic nerve crush injury. Studies aligning animal model investigation with observational data from well-characterized human peripheral neuropathies, including transcriptomics and proteomics, as well as animal model studies using a human clinical trial design should foster the identification of clinically relevant biomarkers and effective targeted treatments with limited addiction potential for chronic neuropathic pain in peripheral neuropathy patients.

## Introduction

Peripheral neuropathies (or peripheral nerve disease) are very common disorders characterized by peripheral nerve axonal degeneration, demyelination or both. Approximately 30 million Americans are affected by peripheral neuropathies with over 100 million patients affected worldwide. A common consequence of peripheral neuropathies is chronic neuropathic pain, estimated to affect up to 20% of people worldwide sometime during their lives (1–6). Peripheral neuropathies and associated chronic neuropathic pain cause disability, significantly reduce productivity and increase the economic burden on national and international health care resources (4, 7–9). The mean annualized costs of neuropathic pain in the United States of America (USA) was ~$27,000 per patient in 2012 (10). With an approximate prevalence of 10% (11), estimated annual costs are ~$900 billion in the USA alone. Trauma is the leading cause of death and disability in the USA. The annual incidence of traumatic injury requiring inpatient hospitalization approximates to 525 per 100,000 population (~1.75 million patients) in the USA alone, with an estimated cost of ~$29.1 billion in 2011 (12). Severe traumatic peripheral nerve injury may occur in up to 2.8% of polytrauma patients (13). Based on published data from the USA National (Nationwide) Inpatient Sample, the annual incidence of upper and lower limb peripheral nerve injury is ~57 cases per million with annual direct cost of ~$1 billion (14, 15). Traumatic peripheral nerve injury results in an estimated yearly economic burden of $150 billion in the USA alone attributed to patient disability and extensive use of health care resources, loss of employment, and significant reduction in productivity owing to residual motor and sensory deficits, and chronic neuropathic pain (16, 17). There is a critical need to precisely determine specific cellular and molecular mediators and signaling mechanisms, identify specific molecular therapeutic targets and validate safe treatments for patients with inflammatory and traumatic peripheral neuropathies suffering with chronic neuropathic pain worldwide.

Studies have demonstrated that non-neural cells are important for the initiation and persistence of chronic pain in nerve injury animal models, potentially relevant to human neuropathies. These cells include mast cells, basophils, platelets, monocytes/macrophages, neutrophils, endothelial cells, keratinocytes, and fibroblasts (18). Activation of resident leukocytes and other cell types, and early leukocyte infiltration into damaged tissues are essential to clear up debris as a prerequisite for tissue regeneration. However, their persistence in these tissues could contribute to chronic pain following injury. Chronic pain is linked to signaling molecule release, e.g. cytokines such as tumor necrosis factor α and interleukin-1β, serotonin, histamine, glutamate, adenosine triphosphate, adenosine, substance P, calcitonin-gene related peptide, bradykinin, eicosinoids, prostaglandins, thromboxanes, leukotrienes, endocannabinoids, nerve growth factor, extracellular proteases, and protons (18).

Targeting pro-inflammatory mediators provides an avenue to effectively treat chronic neuropathic pain (19, 20). Cytokine neutralizing therapies have successfully translated as effective therapies for several rheumatologic disorders. Although significant information on cytokine signaling has been obtained from different chronic pain animal models, precise data from patients is relatively lacking. A recent study demonstrated increased expression of 17 cytokines/chemokines that were significantly up- or down-regulated in severe neuropathic pain patients compared to healthy controls (8 from saliva, 7 from plasma and 2 from cerebrospinal fluid). Correlation analysis showed that the most important proteins linked to pain intensity were found in plasma (21). A major challenge is that these molecules are widely expressed in tissues throughout the body with multiple biological effects, and are rapidly upregulated as part of the innate immune response to injury, infection and inflammation. Systemic modulation of pro-inflammatory molecular signaling to treat chronic neuropathic pain could result in deleterious adverse effects. However, specific cytokine expression in plasma or saliva could serve as disease activity biomarkers. A small pilot study suggested that YKL-40, also known as chitinase-3-like protein 1, combined with chemokine CCL3 may serve as a biomarker in chronic neuropathic pain (21).

Chemokines (‘chemotactic cytokines’) have been of particular interest in chronic pain disorders as these molecules, through their binding to their G protein-coupled receptors, can trigger intracellular signaling pathways that facilitate direct cellular infiltration, proliferation, survival, and inflammation under homeostatic and pathological conditions (22, 23). Multiple chemokine-chemokine receptor pairs expressed in the peripheral and central nervous systems have been implicated in mediating chronic neuropathic pain *via* enhancing neuroinflammation. Targeting specific chemokine/chemokine receptors by small interfering RNAs, blocking antibodies, or small molecule antagonists seems attractive therapeutically for chronic neuropathic pain (22, 23). Unfortunately there has been a failure to translate the wealth of knowledge on chemokine signaling during neuroinflammation and chronic pain into effective therapeutics in patients. Chemokine system redundancy, promiscuity, variable expression profiles dependent on disease phase or time course, and expression of multiple chemokines that participate in the delicate balance between pro-inflammatory and anti-inflammatory signaling following tissue injury and repair provide some reasons for clinical translational failure.

Leukocyte trafficking into peripheral nerves, dorsal root ganglia (DRG) and spinal cord has been implicated in chronic neuropathic pain pathogenesis (24, 25). Leukocyte trafficking across microvessels from circulating blood into tissues is a sequential, coordinated process involving specific selectins, chemokines and adhesion molecules on microvascular endothelial cells, and selectin counterligands, chemokine receptors, integrins and matrix metalloproteases expressed by leukocytes (26–31). Peripheral nerve leukocyte infiltration is pathogenically relevant in inflammatory neuropathies based on observational data from untreated patient nerve biopsies and is associated with disease progression based on data derived from representative murine models (32, 33). There is emerging data that specific leukocyte subpopulations infiltrate into the peripheral nerve endoneurium (i.e. the innermost peripheral nerve compartment where all axons reside) in the commonly studied chronic constriction injury and partial ligation peripheral neuropathy models (25, 34, 35). Specific chemokines expressed on activated microvascular endothelial cells can attract and bind to leukocytes expressing selective chemokine receptors. This interaction induces a conformational change in selected leukocyte integrins (formed by non-covalently bound heterodimers consisting of α and β subunits), converting them from an inactive non-binding state to an active binding state that engage selective endothelial cell adhesion molecules causing firm leukocyte adhesion prior to transmigration into injured tissues (26, 30, 36–38).

CD11b, also known as α_M_-integrin (ITGAM), forms a heterodimer with CD18 (also known as β_2_-integrin). CD11b-CD18 (also known as Mac-1 or complement receptor-3) is expressed predominantly by myeloid cells such as monocytes/macrophages, neutrophils and dendritic cells and a subset of activated T-cells. CD11b-CD18 binds to a wide variety of ligands, including those of the intercellular adhesion molecule (ICAM) family, particularly ICAM-1, the complement protein iC3b, fibrinogen, heparin and laminin, and is active in the innate and adaptive immune responses. The approximately 200-peptide residue α_M_I-domain within CD11b mediates ligand binding and is responsible for its broad substrate specificity (39–42). Depending on the disease and disease state, CD11b may have pro-inflammatory or anti-inflammatory functions (32, 43, 44). This provides the impetus to correlate its biological function during the time-course of specific disorders. CD11b-ICAM-1-mediated monocyte trafficking has been implicated in pathologic peripheral nerve inflammation (32, 43, 45–48). There is data demonstrating strong associations between peripheral nerve monocyte recruitment and neuropathic pain behaviors in mouse peripheral neuropathy models, with several studies indicating that modulation or attenuation of monocyte recruitment into nerves is associated with reduced nociception in rodents (24, 25, 34, 49–52). The high molecular recognition specificity of the CD11b α_M_I-domain (41) would support the design of systemically active antagonists (e.g. humanized mouse monoclonal antibodies) that selective block high affinity ICAM-1 dependent monocyte and neutrophil trafficking without crossing the blood-brain and blood-spinal cord barrier to modulate microglial function or affect phagocytic and other innate immune responses that are dependent on ligand binding to other peptide residues within the domain.

We have studied murine models of peripheral nerve inflammation and axonal injury that recapitulate essential clinical/neurobehavioral, motor electrophysiological and histopathological features of selected human peripheral neuropathies, guided by observational data obtained from patients (32, 33, 53–57). The major emphasis in demyelinating neuritis and axonal injury research has been on motor recovery with less emphasis on pain resolution, despite the fact that treated patients commonly develop refractory debilitating chronic neuropathic pain (28). Pain research using rodent peripheral nerve injury models such as the sciatic nerve partial ligation or chronic constriction injury has resulted in the discovery of neuronal, glial, molecular, and genetic mechanisms involved in nociception (58–60); however, these models may not fully represent the repertoire of pathologic changes in specific human peripheral neuropathies. Due to lack of objective and reliable chronic pain biomarkers, patient pain assessment scales rely on subjective verbal reports/questionnaires that cannot be obtained from laboratory animals for correlative studies.

Glial-derived neurotrophic factor (GDNF), a mitogen secreted by Schwann cells in peripheral nerves, promotes mammalian peripheral nerve or nerve root axonal regeneration and resolution of pain behaviors after injury *in vivo* (54, 61, 62). Unfortunately, GDNF and its receptor agonists have failed to translate as safe and effective analgesic drugs for peripheral neuropathies (63). However, mixed strain Tamoxifen-inducible *Gdnf* conditional knockout 129SvJ: C57BL/6 mice may develop chronic nociception behaviors following non-transecting sciatic nerve crush injury compared to wildtype mice (54, 64). Studying putative mechanisms of inflammation and pain behaviors in Tamoxifen-induced *Gdnf* conditional knockout mice with persistent axonal degeneration, blood-nerve barrier permeability, chronic nociception and impaired axonal regeneration after peripheral nerve injury provides a reliable model to study traumatic neuropathies with pathologic findings similar to nerve biopsies of axonal peripheral neuropathic patients with chronic pain in clinical practice. This model may provide insights pertaining to intraneural cytokine expression at different stages of the nerve injury-nerve repair-nociception continuum that could support pathogenic mechanism hypothesis generation or chronic neuropathic pain biomarker discovery in axonal neuropathy patients.

The severe murine experimental autoimmune neuritis (sm-EAN) model of Guillain-Barré syndrome, and the spontaneous autoimmune peripheral polyneuropathy (SAPP) model of chronic inflammatory demyelinating polyradiculoneuropathy (CIDP) provide experimental tools to evaluate the correlation between pathogenic peripheral nerve leukocyte infiltration and acute and chronic restricted peripheral nerve demyelinating neuropathies with associated residual or chronic pain respectively. The ultimate goal is to better understand the spatial and temporal relationships between cytokine expression, leukocyte infiltration and chronic nociception, and discover safe and more efficacious treatments for chronic neuropathic pain associated with inflammatory and traumatic neuropathies that do not result in addiction, tolerance or dependence. Disease modifying drugs that limit inflammation, restore endoneurial homeostasis at the blood-nerve barrier and promote axonal regeneration in addition to effective analgesia would be ideal. Our initial work using these models of inflammatory and traumatic neuropathy suggests an important role for CD11b+ leukocyte infiltration in chronic pain pathogenesis, supported by observational human peripheral neuropathy data. CD11b antagonism can modulate chronic nociception without inducing addictive behaviors in adult mice.

## Materials and methods

Our research mice were housed in the Research Services Building, University of Alabama at Birmingham (UAB), Birmingham, United States of America in a specific dedicated pathogen-free room maintained at 24°C. All mice were kept in micro-isolator cages with chow and water provided *ad libitum*, maintaining a 12 hour light–dark cycle with standard environmental enrichment without restrictions to free movement. Research studies received approval from the UAB Institutional Animal Care and Use Committee (Protocol # 141009958, 141009959 and 141009960), and were conducted in accordance with the Animal Welfare Act, the U.S. Government Principles Regarding the Care and Use of Animals, the Guide (Guide for the Care and Use of Laboratory Animals), National Research Council, Institute of Laboratory Animal Resources, the Public Health Service Policy on Humane Care and Use of Laboratory Animals and the additional local policies of the UAB Center of Comparative Medicine.

### Mice models

#### Sm-EAN in female SJL/J mice

We induced sm-EAN (a representative animal model of Guillain-Barré syndrome in which brain and spinal cord inflammation are absent histologically) by subcutaneous injection of bovine peripheral nerve myelin emulsified in complete Freund adjuvant in the backs of 8-12 week old female SJL/J mice. Intraperitoneal pertussis toxin and recombinant mouse interleukin-12 administered were given as co-adjuvants, as previously published (32, 33, 57). Age-matched female SJL/J mice receiving recombinant mouse interleukin-12 and pertussis toxin without subcutaneous bovine peripheral nerve myelin inoculation were used as controls. Sm-EAN has a 100% disease incidence in female SJL/J mice, with male mice being resistant at this age group. At pre-defined time points from day 0-65 post-induction, sciatic nerves were harvested under deep ketamine-xylazine anesthesia and immediately cryopreserved in Tissue-Tek^®^ optimum cutting temperature (OCT) compound at -80°C for future histological processing.

#### SAPP in CD86-deficient non-obese diabetic (NOD) mice

SAPP is a chronic demyelinating neuritis that occurs in female B7-2 (CD86)-deficient non-obese diabetic mice (NOD.129S4-Cd86tm1Shr/JbsJ; initially purchased from the Jackson Laboratory [Bar Harbor, ME]), with features that recapitulate a severe form of progressive CIDP with subsequent secondary axonal loss. Disease onset is at 20 weeks of age with 100% of females reaching maximum severity by 32 weeks that persists >40 weeks of age (55, 65). ~20-30% of male mice develop SAPP and were not studied due to unpredictable disease onset and phenotype. These mice do not develop diabetes due to the germline CD86 knockout (53, 55, 65). At pre-defined time points between 20-40 weeks of age, sciatic nerves were harvested under deep ketamine-xylazine anesthesia and immediately cryopreserved in OCT compound at -80°C for future histological processing. To determine whether nociception occurs at the time of SAPP disease onset, 18-22 week old female mice were studied.

#### Sciatic nerve crush injury in Tamoxifen-inducible conditional *Gdnf* knockout (*Gdnf* CKO) mice

*Gdnf* ^Flox/Flox^ mice (129/SvJ: C57BL/6, containing LoxP flanking sequences on *Gdnf* exon 3) and *Gdnf* ^LacZ/+^ were obtained *via* a Materials Transfer Agreement from Instituto de Biomedicina de Sevilla (IBiS), Seville, Spain. Due to suboptimal Cre recombinase-mediated *Gdnf* deletion in *Gdnf* ^Flox/Flox^ mice, heterozygous *Gdnf* ^LacZ/+^ C57BL/6 mice were crossed with the commercially available hemizygous Esr1-Cre recombinase C57BL/6 transgenic mice [B6.Cg-Tg(CAG-cre/Esr1*)5Amc/J, Strain #: 004682, The Jackson Laboratory)] referred to as Esr1-cre/+ to generate *Gdnf* ^LacZ/+^; Esr1-Cre/+ mice. These mice were crossed with *Gdnf* ^Flox/Flox^ mice to generate Tamoxifen-inducible GDNF^LacZ/Flox^; Esr1-Cre/+ mice (*Gdnf* CKO) and control littermates (wildtype [*Gdnf* WT]: *Gdnf* ^Flox/+;^ +/+ and heterozygous [*Gdnf* HET]: *Gdnf* ^LacZ/Flox^; +/+) mice for subsequent experiments (54, 66).

In order to study a traumatic peripheral neuropathy characterized by axonal degeneration with increased blood-nerve barrier permeability, we performed non-transecting sciatic nerve crush injury on age- and sex-matched adult (>12 weeks old) *Gdnf* WT, *Gdnf* HET and *Gdnf* CKO mice at least 4 weeks following Tamoxifen injection, as previously published (54). Complete axonal regeneration occurs between 4-8 weeks after sciatic nerve injury in genetically normal mice (67, 68). At pre-defined time points 0-14 days after nerve injury, sciatic nerves and L5 lumbar dorsal root ganglia (DRG) were harvested under deep ketamine-xylazine anesthesia and immediately cryopreserved in OCT compound at -80°C for future histological processing. To determine whether chronic nociception develops in Tamoxifen-inducible *Gdnf* CKO mice with known persistent axonal degeneration, blood-nerve barrier permeability and impaired axonal regeneration (54), equal numbers of male and female *Gdnf* WT and *Gdnf* CKO were studied 4-6 weeks after crush injury. *Gdnf* CKO mice demonstrated abrogated sciatic nerve Gdnf expression after injury relative to uninjured (Sham control) nerve basal expression (~1.6-fold) compared to *Gdnf* WT mice with a ~7.5-fold relative increase in sciatic nerve Gdnf expression 7 days after injury relative to Sham control nerves (54).

### Reflexive neurobehavioral nociception assays

Prior to performing neurobehavioral nociception assays, we placed each mouse in a Plexiglass chamber situated atop a Plexiglass platform and allowed it to acclimate for an hour. In order to evaluate mechanical hypersensitivity, we performed von Frey filament application using a simplified up-down method (69). In summary, we applied a calibrated polypropylene monofilament (North Coast Medical) from below to the plantar surface of each hind paw and recorded the force at which the hind paw was withdrawn to study mechanical hypersensitivity. We used filaments numbered 2 through 9, always starting with filament 5. The testing progressed up or down, such that a positive withdrawal response to a particular filament indicated the next lower value filament for use in the subsequent test, while a negative non-withdrawal response indicated the next higher filament be used. Each hind paw was evaluated two times per mouse. We calibrated the von Frey filaments after each series of experiments and converted the paw withdrawal latencies to force (in grams), using previously published methods (69). The average force in each paw was used for comparative analyses.

To study thermal hyperalgesia, we utilized the published Hargreaves method (70). We placed a moveable infrared generator connected to an automatic timing device (Ugo Basile) below the plantar surface of each hind paw and recorded the paw withdrawal latency (in seconds) after the stimulus. To evaluate cold allodynia, we performed the cold plantar assay (71), as previously published. We applied a cold probe consisting of a compacted powdered dry ice pellet to each hind paw plantar surface and manually recorded the paw withdrawal latency (in seconds) using a digital stopwatch. We performed these tests twice in each hind paw and the average withdrawal latency used for comparative analyses.

In the sciatic nerve crush injury model, we calculated mean nociception withdrawal thresholds of the injured side relative to the uninjured side (Sham control) to limit the effect of biological variability in reflexive nociception between mice, as each mouse serves as its own control. When analgesic drugs were systemically administered, the mean absolute latency from injured side only per mouse was used for comparative analyses to limit potential non-specific effects on cutaneous perception on the uninjured side. In the SAPP model (characterized by chronic demyelinating neuritis that affects fore and hind limbs), the mean absolute withdrawal latency from both hind limbs was used per mouse for comparative analyses.

### Indirect fluorescent immunohistochemistry

Archived cryopreserved sciatic nerve biopsies and lumbar L5 DRG from our murine models of inflammatory and traumatic neuropathies stored in OCT compound at -80°C, were studied to evaluate intraneural CD11b leukocyte infiltration by indirect fluorescent immunohistochemistry, with appropriate controls. In order to demonstrate potential translational relevance, we also performed indirect fluorescent immunohistochemistry to detect endoneurial CD11b+ leukocytes in chronic neuropathic pain patients, indicated by the validated modified Wong-Baker (WB) Faces Pain Rating scale (0-10, with 0 meaning no pain and 10 the worst pain imaginable) (72). The archived frozen sural nerve biopsies from three different peripheral neuropathy patients (one each with CIDP, human immunodeficiency virus (HIV)-associated distal sensory axonal polyneuropathy and primary vasculitic axonal neuropathy) were obtained from the Shin J. Oh Muscle and Nerve Histopathology Laboratory at UAB. These specimens had been stored in OCT compound at -80°C in the clinical laboratory’s repository. Permission to use archived de-identified clinical specimens for biomedical research (which does not constitute human subject’s research) was approved by the UAB Institutional Review Board (protocol exemption # 140321012).

Serial 10 µm thick axial cryostat mouse sciatic nerve and DRG, and consecutive 20 µm thick axial cryostat human sural nerve sections were processed to determine intraneural CD11b expression, using previously published protocols (33, 55, 57). Sections were fixed in acetone at -20°C, washed and air-dried prior to blocking with 10% normal goat serum in 1X PBS. All primary and secondary antibodies were diluted in 2% normal goat serum in 1X PBS. The following primary antibodies were used: rat anti-mouse/human CD11b IgG2b (clone M1/70, Bio X Cell, catalog # BE0007: 5 μg/mL) and polyclonal rabbit anti-mouse CD45 IgG (Abcam, catalog # ab10558: 1:150), with secondary antibodies goat anti-rat IgG (H+L) Alexa Fluor^®^ 488 and 594 conjugates and goat anti-rabbit IgG (H+L) Alexa Fluor^®^ 594 conjugate (all from ThermoFisher Scientific: 4 µg/mL). All sections were stained with 0.45 μM 4’, 6-diamidino-2-phenylindole (DAPI) for 5 minutes to detect nuclei and mounted with ProLong^®^ Gold antifade mounting medium (Life technologies). Photomicrographs were taken using an Eclipse Ci-S Upright epifluorescent microscope with a D5-Qi2 camera (Nikon Instruments Inc., Melville, NY). Automated quantification of CD11b+ leukocytes was blindly performed on merged photomicrographs using the Nikon NIS-Elements AR software. Data were expressed as number of cells/section in each mouse to eliminate errors in cross-sectional area that may occur with tissue processing *ex vivo* and variable increases in endoneurial area that may occur with inflammation or tissue injury edema. Representative photomicrographs were merged using Adobe Photoshop and mounted into a single figure using Microsoft PowerPoint, converted to high quality print PDF prior to final image conversion with Adobe Photoshop.

### CD11b antagonism

To evaluate the effect of CD11b antagonism in treating a traumatic neuropathy potentially associated with chronic neuropathic pain, we administered 5 mg/kg of a function-neutralizing depleting rat anti-mouse CD11b IgG2b monoclonal antibody (clone M1/70, Bio X Cell) *via* intraperitoneal (IP) injection to male and female Tamoxifen-induced *Gdnf* CKO and WT mice on days 13-17 following unilateral sciatic nerve crush injury to inhibit persistent sciatic nerve CD11b+ leukocyte infiltration, using previously published methods (32). An isotype control antibody was administered similarly to age- and sex-matched mice. We performed validated reflexive neurobehavioral nociception tests for cold allodynia and thermal hyperalgesia in a blinded manner at predefined intervals for up to 26 days after the last antibody injection (i.e. day 43 post-injury), as previously described.

### Conditioned place preference assay

To evaluate whether effective CD11b antagonism that may reduce chronic nociception causes drug reward potential behaviors in mice that could hinder the translation of systemic CD11b antagonists in chronic neuropathic pain patients with peripheral neuropathies, we performed a validated conditioned place preference (CPP) test in 4 healthy adult male and female SJL/J mice >12 weeks old per group (73, 74). Female SJL/J mice develop sm-EAN and we had previously published that early CD11b antagonism after disease onset effectively treated sm-EAN by abrogating acute demyelinating neuritis (32). We administered 5 mg/kg IP rat anti-mouse CD11b IgG2b monoclonal antibody (clone M1/70) in a specific chamber followed by normal saline in the other chamber. Age- and sex-matched mice were either treated with 5 mg/kg IP isotype antibody (negative control) or Buprenorphine 0.1 mg/kg SC (positive control) followed by equal volumes of normal saline. We measured the total time spent in either chamber by automated video analysis for each mouse, and averaged data from each treatment group for comparative analysis.

### Mice cytokine protein array

To decipher cytokine expression profiles that may discriminate between mice that develop chronic nociception from those who fully recover after sciatic nerve crush injury, we blindly performed cytokine protein arrays (Mouse Inflammation Antibody Array C1, RayBiotech catalog # AAM-INF-1) on whole sciatic nerve homogenates from age- and sex-matched *Gdnf* CKO and *Gdnf* WT mice on day 7 after unilateral crush injury, with uninjured nerves (Sham surgery) from the same mice serving as internal controls (54). Day 7 was chosen for the assay as we had demonstrated that early recovery from injury occurred at this time point which coincided with maximal sciatic nerve Gdnf expression. We pooled 4 sciatic nerves (2 females and 2 males) from each experimental group, extracted protein from homogenates as previously published (54, 57), and processed protein arrays concurrently according to the vendor’s protocol. Arrays were imaged together using a Bio-Rad ChemiDoc™ XRS imaging system. Digital images analyzed by semi-quantitative 2-D spot densitometry to determine relative protein expression with the AlphaEaseFC software program (ProteinSimple, San Jose, CA).

### Statistical analysis

Statistical analyses were performed using Prism software (GraphPad Software, Inc.; La Jolla, CA). One- or two-tailed paired or unpaired Student’s/Welch’s t-test or Mann Whitney U test was used for comparison between experimental groups based on the Shapiro-Wilk test of normality (including measures of skew and kurtosis). Holm-Sidak’s test was used to correct for multiple variable comparisons. Means are displayed for each parameter evaluated as a bar histogram, with individual data points shown to illustrate data spread as indicated. p<0.05 was indicative of statistical significance.

## Preliminary Results

### Chronic nociception occurs in Tamoxifen-induced *Gdnf* CKO adult mice following unilateral sciatic nerve crush injury

**Figure 1:**
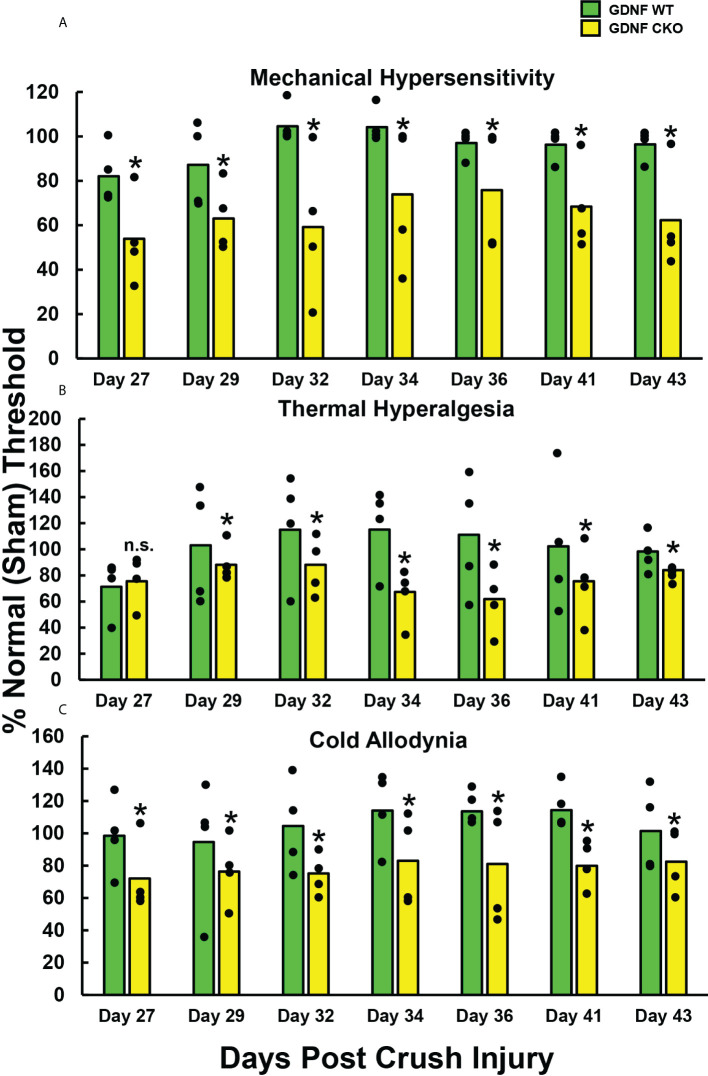
Chronic reflexive neurobehavioral nociception in adult *Gdnf* CKO and WT mice after unilateral sciatic nerve crush injury. Bar histograms demonstrate mean reflexive paw withdrawal thresholds from the same side as injured sciatic nerves relative to paw withdrawal thresholds from the uninjured (Sham control) side, expressed as % of the uninjured side. Statistically significant reductions in relative paw withdrawal thresholds to mechanical hypersensitivity **(A)**, thermal hyperalgesia **(B)** and cold allodynia **(C)** are observed in *Gdnf* CKO mice compared to age- and sex-matched *Gdnf* WT mice from day 29 following sciatic nerve crush injury for all modalities that persist up to 43 days post-injury, with significant differences seen with mechanical hypersensitivity and cold allodynia from day 27. *p < 0.05. N = 4.

To determine whether persistent axonal degeneration and delayed axonal regeneration, commonly observed in sural nerve biopsies of chronic peripheral neuropathic pain patients, is associated with chronic nociception in mice, we quantified mechanical hypersensitivity (A), thermal hyperalgesia (B) and cold allodynia (C) withdrawal thresholds in age- and sex-matched adult *Gdnf* CKO and *Gdnf* WT mice from days 27-43 after sciatic nerve crush injury, using well-established protocols as previously described (69–71). Significantly reduced mean % withdrawal thresholds (relative to the uninjured [Sham] nerves in each mouse) were observed in *Gdnf* CKO compared to *Gdnf* WT mice from 27-29 days post-injury that persisted until day 43 (**Figure 1**). Normal thresholds (~100%) were observed in *Gdnf* WT mice by 32 days post-injury, indicative of complete nociception resolution in mice with normal sciatic nerve Gdnf expression after crush injury (64). These data support the notion that Tamoxifen-induced *Gdnf* CKO mice can be used to study cellular and molecular determinants and signaling mechanisms of chronic nociception following unilateral sciatic nerve injury.

### Spontaneous Autoimmune Peripheral Polyneuropathy (SAPP) onset is associated with increased reflexive neurobehavioral nociception

**Figure 2:**
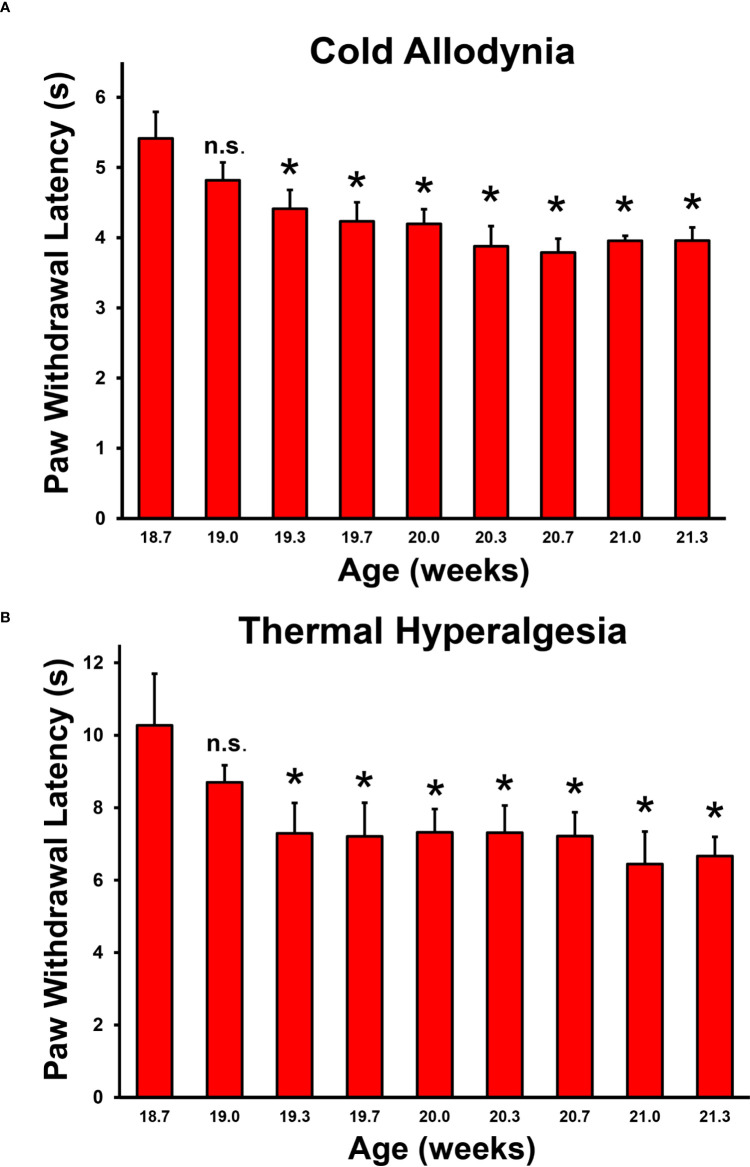
Baseline and disease reflexive neurobehavioral nociception in adult female CD86-deficient NOD mice. Bar histograms from a representative experiment demonstrate mean paw withdrawal latencies (in seconds) testing for cold allodynia **(A)** and thermal hyperalgesia **(B)** prior to and around the expected onset of chronic demyelinating neuritis. Statistically significant mean reduced paw withdrawal latencies for cold allodynia **(A)** and thermal hyperalgesia **(B)** are observed in this spontaneous chronic demyelinating neuritis model from 19.3 weeks of age, at the approximate known onset of chronic inflammation, demyelination and weakness (20 weeks of age). Error bars indicates standard error of the mean *p < 0.05. n.s. not significant, N = 6.

We sought to determine whether nociception occurs at disease onset when the earliest signs of weakness and demyelinating neuritis also occur, and persists in female CD86-deficient NOD mice that reliably develop SAPP (55, 65). We blindly performed longitudinal reflexive neurobehavioral nociception tests (cold allodynia and thermal hyperalgesia) 3 times a week for 4 weeks, using published methods as described previously (70, 71). We observed significantly reduced mean paw withdrawal thresholds from 19.3 weeks of age (**Figure 2**), with 20 weeks being the expected SAPP disease onset age based on muscle weakness scores (55, 65). These data support the notion that nociception occurs in SAPP-affected mice at disease onset, providing an avenue to better understand early cellular and molecular determinants, and signaling mechanisms of chronic nociception associated with chronic demyelinating neuritis.

### Increased sciatic nerve CD11b+ CD45+ leukocyte infiltration is associated with motor disease severity in murine inflammatory and traumatic peripheral neuropathy models

**Figure 3:**
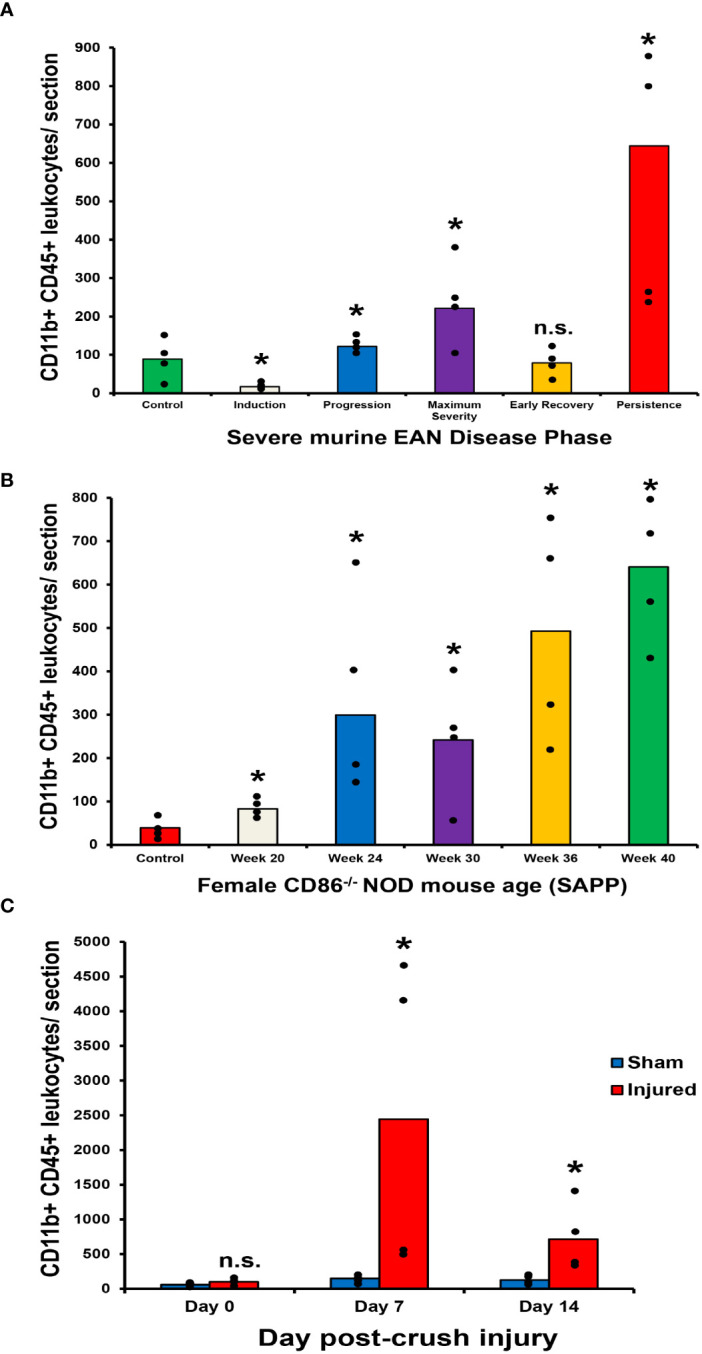
Sciatic nerve CD11b+ CD45+ leukocyte infiltration in murine peripheral neuropathy models. Bar histograms depict the mean endoneurial sciatic nerve CD11b+ CD45+ leukocyte counts per axial section (400X magnification) relative to disease course in sm-EAN **(A)**, time (in weeks) in SAPP **(B)** and time (in days) following unilateral crush injury **(C)** are shown. In sm-EAN **(A)**, disease induction occurs within the first 7 days following bovine peripheral nerve myelin inoculation, disease progression between days 7-21 and maximum severity between days 26-32. Early recovery occurs between days 33-40 post-inoculation with full recovery expected between days 55-65. Disease persistence refers to mice with appreciable muscle weakness on day 65 post-inoculation. Days 0, 15, 30, 37 and 65 post-inoculation are studied to represent these disease phases respectively. Statistically significant increases in mean CD11b+ CD45+ leukocyte counts are seen during sm-EAN disease progression, at maximum severity and with disease persistence relative to controls **(A)**. In SAPP, spontaneous disease onset is at 20 weeks of age and weakness persists throughout the life of affected mice. Significant increases in mean CD11b+ CD45+ leukocyte counts are seen relative to control mice without SAPP throughout the disease course **(B)**. Following unilateral sciatic nerve crush injury, significant differences in mean CD11b+ CD45+ leukocyte counts are seen on day 7 (at the time of maximal sciatic nerve Gdnf expression) and day 14 (during the early recovery phase associated with restoration of blood-nerve barrier impermeability and early axonal regeneration in WT mice) compared to uninjured (Sham control) nerves from the same mice **(C)**. *p < 0.05. n.s. not significant, N = 4.

Guided by previous studies implicating pathogenic CD11b+ leukocyte infiltration in peripheral neuropathies with chronic pain (34, 35, 52), we evaluated the longitudinal expression of CD11b+ leukocytes in murine models of inflammatory and traumatic neuropathies. Using previously archived cryopreserved sciatic nerves from adult female mice with sm-EAN (with known previously published detailed characterization of motor disease phases and severity) (32, 33, 57), we observed increased CD11b+ CD45+ leukocyte infiltration with worse motor progression/severity. Reduced numbers were observed in mice with early motor recovery. Further increases were seen in mice with persistent weakness 65 days after disease induction (**Figure 3A**). Similarly, adult female CD86-deficient NOD mice with SAPP showed increased CD11b+ CD45+ leukocyte infiltration with age-dependent progressive weakness SAPP (53, 55), relative to age-matched wildtype NOD mice (control) without SAPP (**Figure 3B**). In the sciatic nerve crush injury traumatic neuropathy model (54), we observed intense injured sciatic nerve CD11b+ CD45+ leukocyte infiltration in both male and female *Gdnf* WT and *Gdnf* CKO mice at the peak of axonal degeneration (day 7 post-injury) relative to the Sham surgery (uninjured) sciatic nerves in the same mice. We observed a reduction in CD11b+ CD45+ leukocyte counts at day 14 when there is demonstrable histological evidence of axonal regeneration (**Figure 3C**). However, higher mean CD11b+ CD45+ counts were measured in *Gdnf* CKO mice with known persistent axonal degeneration and delayed axonal regeneration compared to *Gdnf* WT mice (54, 64) on day 14 (mean 686 vs. 463, p<0.05, data not shown).

**Figure 4:**
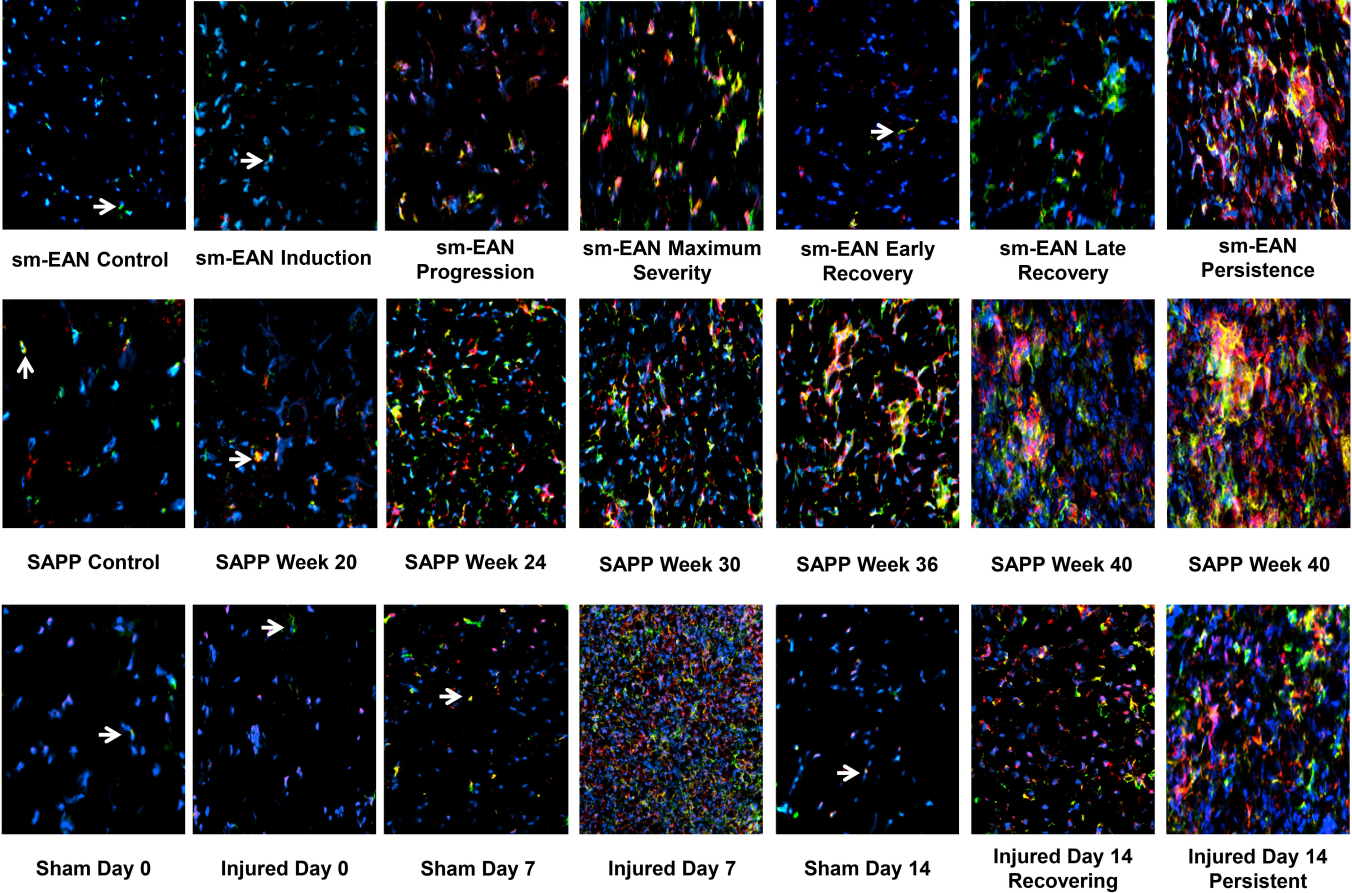
Sciatic nerve CD11b+ CD45+ leukocyte infiltration in mouse inflammatory and traumatic neuropathy models. Representative digital axial photomicrographs of mouse sciatic nerves show rare positive-staining leukocytes (white arrows) in sm-EAN, SAPP, and Sham surgery control nerves. These cells are also rarely seen in sm-EAN mice during early recovery and on day 0 in crush injured sciatic nerves. Infiltration of CD11b+ CD45+ leukocytes (yellow and orange colors) is associated with motor disease progression/severity and persistent axonal degeneration in these adult mouse inflammatory and traumatic peripheral neuropathy models. Blue (DAPI) depicts nuclei. Original magnification 200-400X. N = 4.

These data suggest a pathogenic relationship between sciatic nerve CD11b+ CD45+ leukocyte infiltration and motor disease severity in these murine models. Representative sciatic nerve CD11b+ CD45+ leukocyte infiltration photomicrographs and motor disease stage/severity in these inflammatory and traumatic peripheral neuropathy mouse models are shown in **Figure 4**. Longitudinal studies that directly evaluate whether persistent CD11b infiltration correlates with chronic nociception in these murine models would support the hypotheses that hematogenous CD11b+ leukocyte retention is important for chronic neuropathic pain pathogenesis.

### CD11b+ CD45+ leukocyte infiltration is observed in the lumbar DRG of sciatic nerve injured *Gdnf* CKO mice with persistent axonal degeneration and delayed axonal regeneration after injury

**Figure 5:**
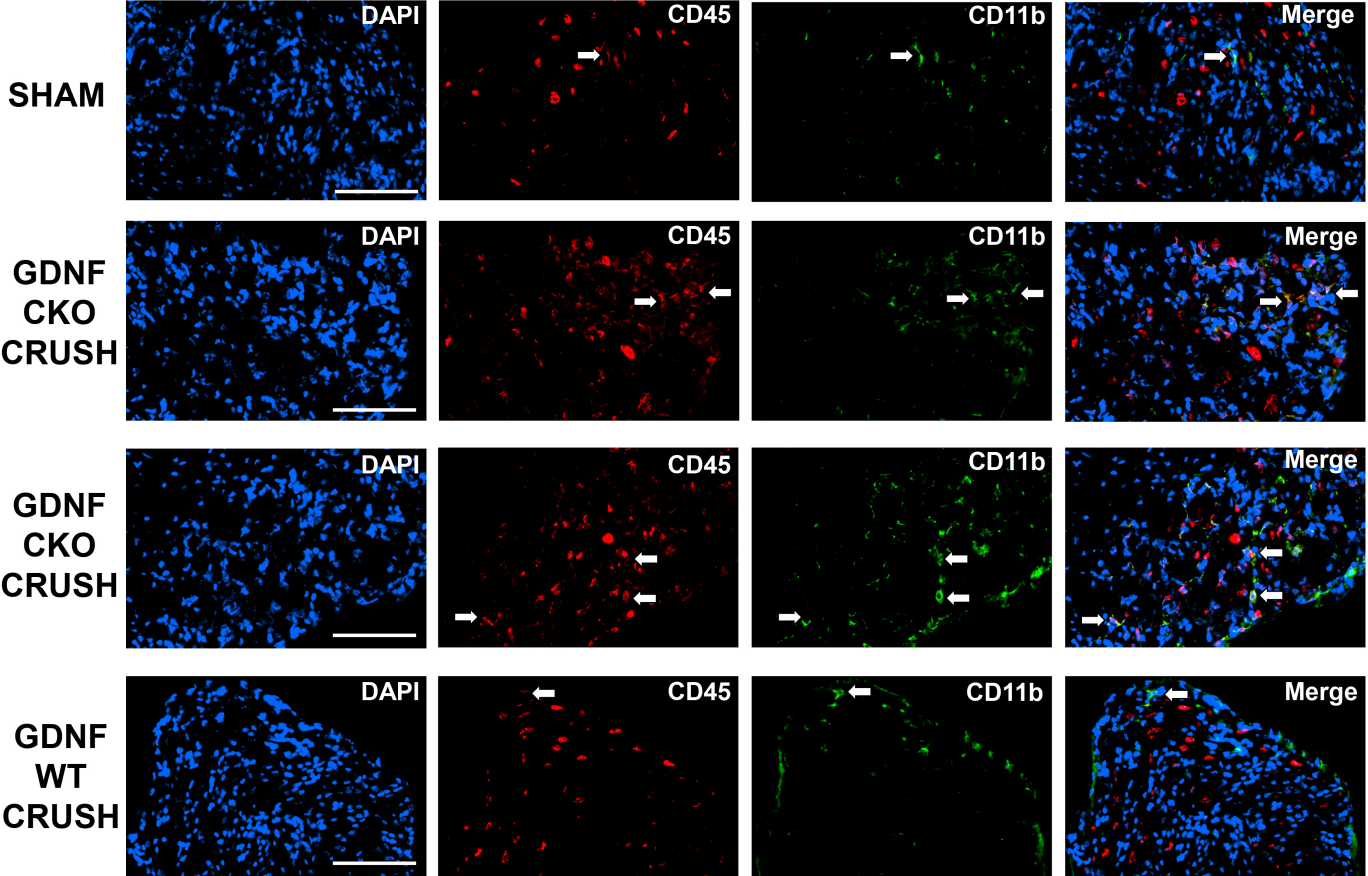
L5 lumbar DRG CD11b+ CD45+ leukocyte infiltration in Tamoxifen-induced *Gdnf* CKO and *Gdnf* WT on day 14 following unilateral sciatic nerve crush injury. Representative digital axial photomicrographs of mouse L5 DRGs show rare positive-staining leukocytes (white arrows) in Sham surgery control L5 DRGs. Sciatic nerve crush injury is associated with increased L5 DRG leukocyte infiltration. More CD45+ leukocytes are seen in *Gdnf* CKO mice compared to *Gdnf* WT mice at a time point associated with expected early axonal regeneration, with higher numbers of CD11b+ CD45+ leukocytes observed. However, CD11b- CD45+ leukocytes are more prevalent in these mice. Scale Bar = 100 µm. N = 4.

We studied the L5 lumbar DRG of age- and sex-matched adult Tamoxifen-induced *Gdnf* CKO and *Gdnf* WT mice following unilateral sciatic nerve crush injury to determine whether lumbar DRG CD11b+ leukocyte infiltration is associated with sciatic nerve axonal recovery, and potentially relevant to overall disease severity by indirect fluorescent immunohistochemistry. We observed increased L5 lumbar DRG CD11b+ CD45+ leukocyte infiltration in *Gdnf* CKO mice on the same side of the sciatic nerve injury (i.e. unilateral) compared to the opposite side (i.e. contralateral) lumbar DRGs, where Sham surgery was performed (**Figure 5**). However, the DRG-infiltrated CD45+ leukocytes were mainly CD11b-. This differs significantly from the injured sciatic nerves where most of the persistent leukocytes were CD11b+. We also observed fewer infiltrated CD45+ leukocytes in *Gdnf* WT mice (associated with early axonal regeneration) compared to *Gdnf* CKO mice (associated with persistent axonal degeneration) (54, 64). As with *Gdnf* CKO mice, most of the DRG infiltrating leukocytes in *Gdnf* WT mice were CD11b-. These data support the hypothesis that persistent CD11b+ leukocyte infiltration in injured peripheral nerves and DRGs may be associated with more severe disease and subsequent development of chronic nociception. We hypothesize that DRG CD11b+ leukocytes may support chronic neuropathic pain development through pro-inflammatory cytokine secretion and activation of nociceptive signaling away from the site of direct nerve injury.

### Endoneurial CD11b+ leukocytes are present in adult peripheral neuropathy patients with chronic neuropathic pain

**Figure 6:**
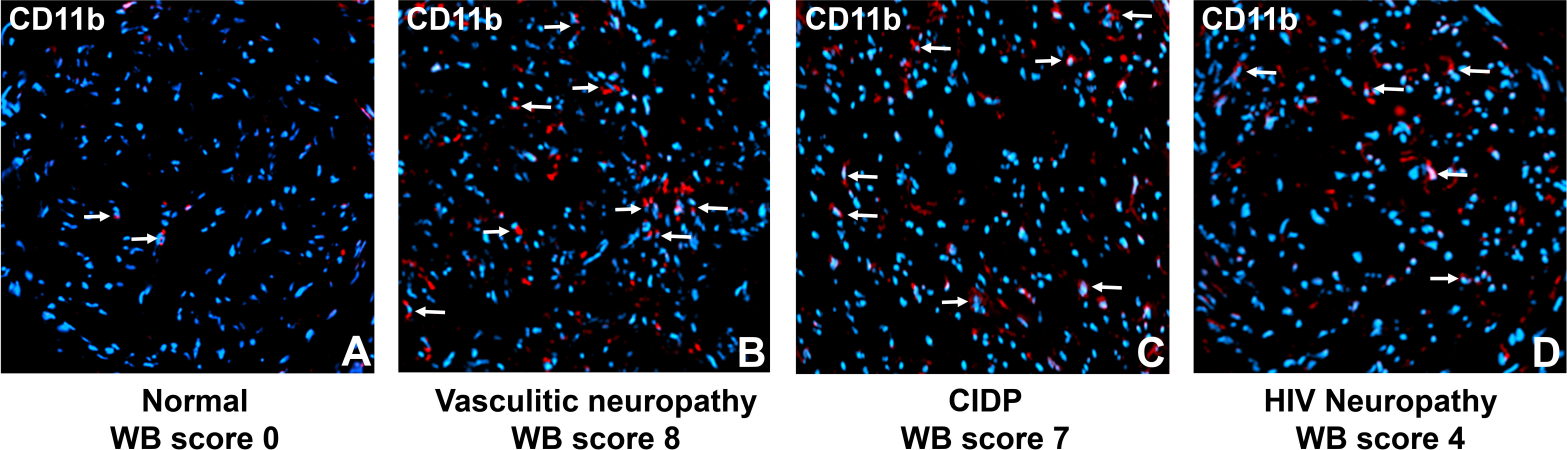
Sural nerve endoneurial CD11b+ leukocytes in peripheral neuropathies associated with chronic pain. Representative digital photomicrographs shows CD11b+ leukocyte expression (white arrows) in three adult patients with chronic pain **(B-D)** contrasted with a histologically normal nerve biopsy **(A)**. Nuclei are shown in blue (DAPI). Original magnification 200X.

We observed rare CD11b+ endoneurial leukocytes in the histologically normal sural nerve, consistent with endogenous macrophages. Increased CD11b+ leukocytes were seen in vasculitic neuropathy, CIDP and HIV neuropathy patients with chronic neuropathic pain (**Figure 6**). These descriptive data suggest that CD11b-mediated leukocyte infiltration is biologically relevant, leading to the hypothesis that targeted therapeutic modulation of CD11b-mediated leukocyte trafficking could effectively treat chronic neuropathic pain associated with associated peripheral neuropathies in patients.

### CD11b antagonism reduces chronic reflexive nociception in mouse traumatic peripheral neuropathy

**Figure 7:**
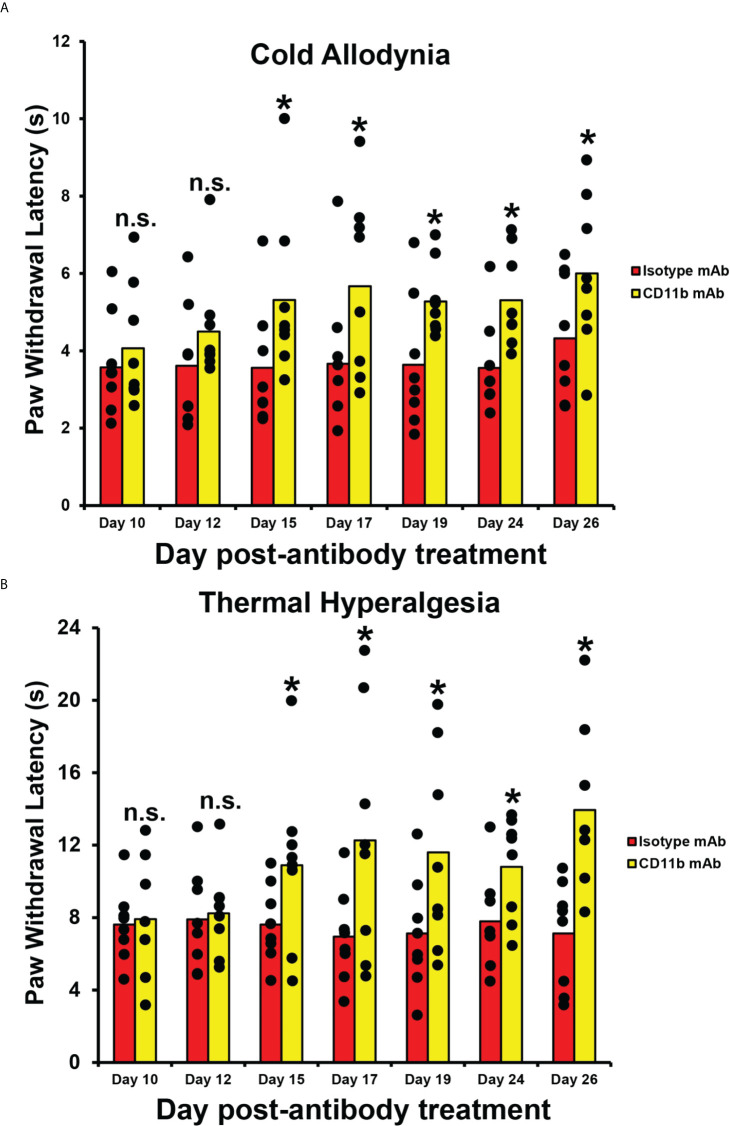
Efficacy of CD11b antagonism on chronic nociception in Tamoxifen-induced adult *Gdnf* CKO mice following unilateral sciatic nerve crush injury. Bar histograms from a representative experiment demonstrate mean paw withdrawal latencies (in seconds) testing for cold allodynia **(A)** and thermal hyperalgesia **(B)** following unilateral sciatic nerve injury and subsequent systemic CD11b antagonism. Significant increases in mean paw withdrawal latencies are observed in mice treated with 5 mg/kg IP of a specific rat anti-mouse CD11b IgG2b monoclonal antibody (CD11b mAb) compared to mice treated with an isotype control antibody (Isotype mAb) from day 15 after last treatment (i.e. day 32 post-injury). Mean uninjured (Sham control) paw withdrawal latency = 5.5 s for cold allodynia **(A)** and 11.9 s for thermal hyperalgesia **(B)**. *p < 0.05. n.s. not significant, N = 8 (4 male and 4 female mice).

To determine whether CD11b antagonism could modulate chronic nociception in mice with persistent axonal degeneration after injury (54, 64), we administered a function neutralizing rat anti-mouse CD11b IgG2b monoclonal antibody to adult *Gdnf* CKO mice 13-17 days after unilateral sciatic nerve crush injury, as previously described. There was a significant persistent increase in mean paw withdrawal latencies in both male and female *Gdnf* CKO mice to cold and hot nociceptive stimulus from day 15 after last treatment (day 32 post-injury) compared to isotype control. This is indicative of reduced chronic nociception in *Gdnf* CKO mice (**Figure 7**). Increased latencies were equivalent to baseline latencies observed in *Gdnf* WT mice. CD11b antagonism at this time interval did not have effect on *Gdnf* WT mean paw withdrawal latencies (data not shown), suggesting sciatic nerve inflammation had resolved prior to treatment in *Gdnf* WT mice. These preliminary studies support the notion that persistent peripheral nerve CD11b+ leukocyte infiltration is pathogenically linked to inflammation and chronic nociception after traumatic nerve injury. Systemic CD11b antagonism using a validated function-neutralizing CD11b monoclonal antibody effectively treated chronic nociception in this model, suggesting potential translational potential to chronic neuropathic pain patients.

### Effective CD11b antagonism with a function-neutralizing monoclonal antibody does not cause drug reward behaviors in healthy adult male and female SJL/J mice

**Figure 8:**
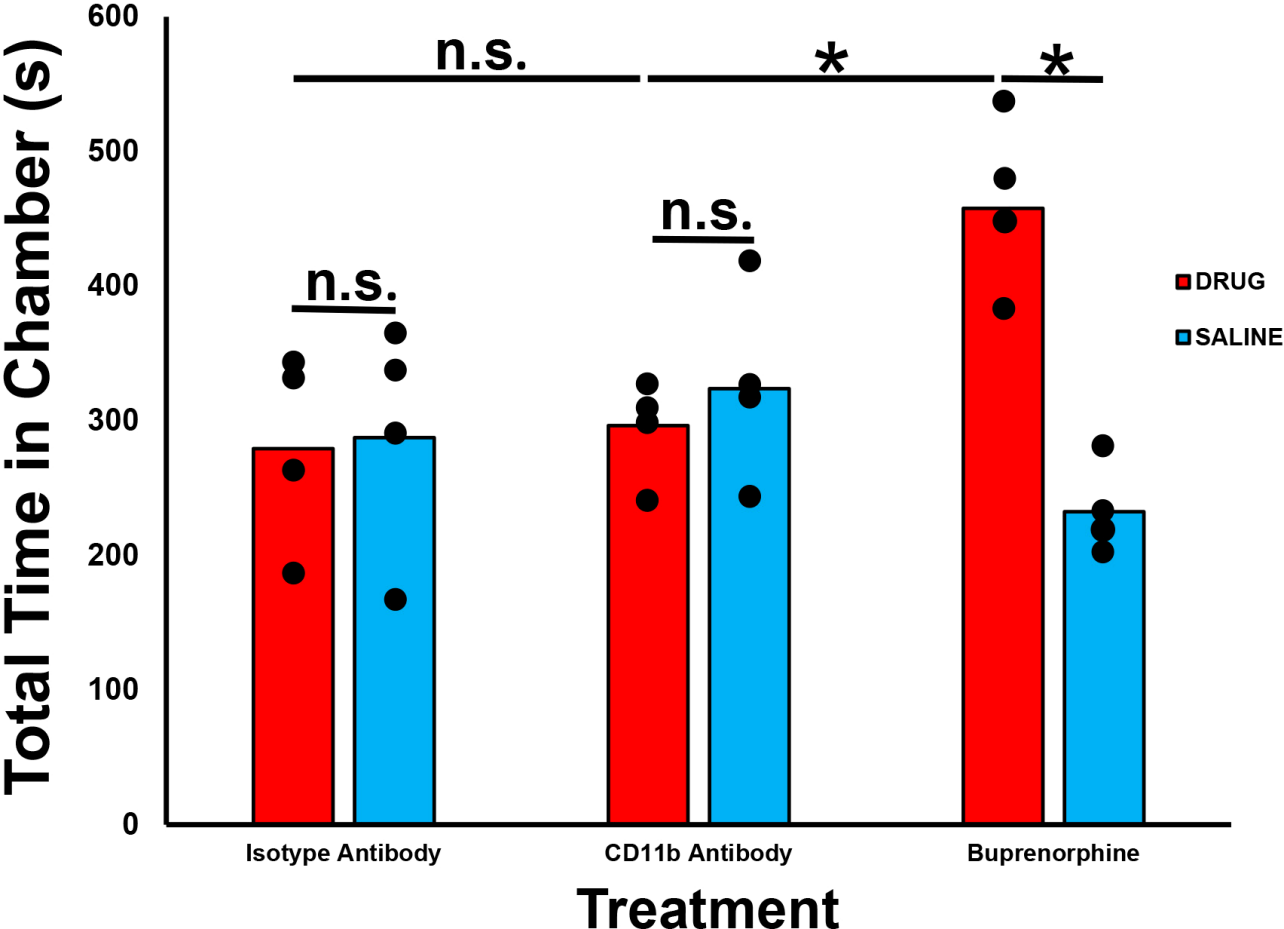
Effect of CD11b administration on drug reward behaviors in healthy adult mice. Bar histograms demonstrating mean total chamber time spent from a representative conditioned place preference test following systemic CD11b blockade are shown. Chamber preference is not seen following CD11b monoclonal antibody administration in adult SJL/J mice relative to saline administration in contrast to significant chamber preference based on total time spent in the drug administered chamber seen following Buprenorphine administration (positive control). Isotype antibody-treated mice serve as a negative chamber preference control. *p < 0.05. n.s. not significant, N = 4.

To determine whether CD11b antagonism could induce drug reward behaviors when given as treatment for chronic nociception, we performed a published validated CPP test in 4 healthy adult male and female SJL/J mice, as previously described. We observed no significant differences in mean time spent in the CD11b antibody treatment chamber compared to the saline treatment chamber. We also observed significantly less time spent in the CD11b antibody treatment chamber compared to Buprenorphine treatment chamber. No differences were observed between the CD11b and isotype antibody treatment chambers (**Figure 8**). These data imply that CD11b antibody antagonism does not cause drug reward potential behaviors in healthy adult SJL/J mice, supporting the rationale to further verify this observation in murine models of inflammatory and traumatic peripheral neuropathies associated with chronic neuropathic pain.

### Expression of sciatic nerve tissue inhibitor of metalloproteinase-1 (TIMP-1) occurs only in the injured sciatic nerves in adult Tamoxifen-induced *Gdnf* CKO during the early recovery phase

**Figure 9:**
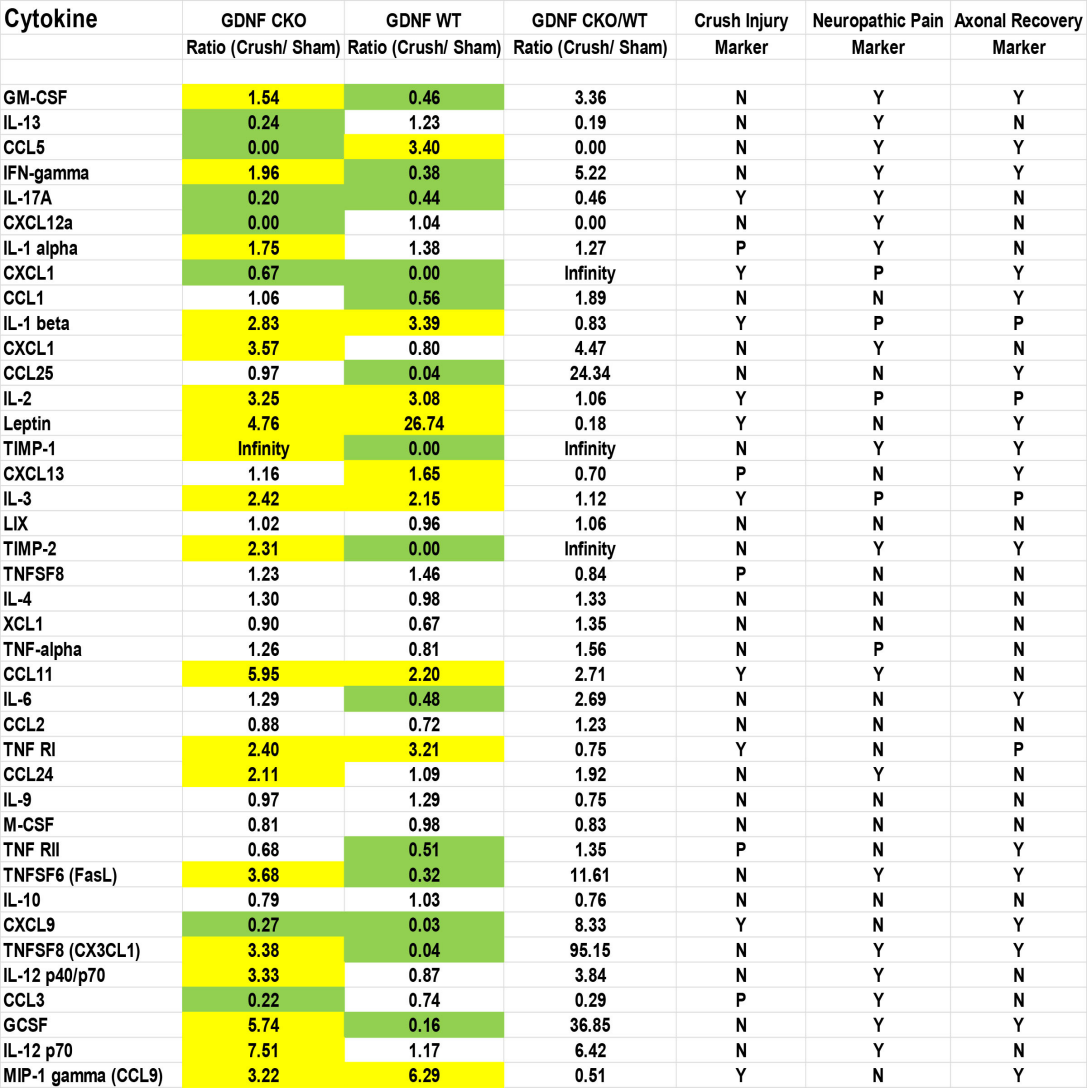
Sciatic nerve cytokine expression profiles in age- and sex-matched *Gdnf* CKO and *Gdnf* WT mice following unilateral crush injury. Whole sciatic nerve homogenate semi-quantitative cytokine array spot densitometry numerical data from a representative experiment show increased (1.5-fold up; yellow) or decreased (1.5-fold down; green) protein expression on day 7 following unilateral crush injury relative to uninjured Sham surgery nerves from the same mice. Maximal Gdnf expression occurs on day 7 following sciatic nerve crush. Cytokines that are increased or decreased in BOTH *Gdnf* CKO and *Gdnf* WT mice could be crush injury biomarkers. Cytokines that are either increased or decreased in *Gdnf* CKO mice with the opposite pattern in *Gdnf* WT mice could be persistent axonal degeneration or chronic nociception predictive biomarkers. Cytokines increased or decreased in *Gdnf* WT mice and the opposite pattern detected in *Gdnf* CKO mice could be predictive biomarkers of early axonal regeneration. N = No, Y = Yes, P = Possible. N = 4 (16 sciatic nerves in total).

**Figure 10:**
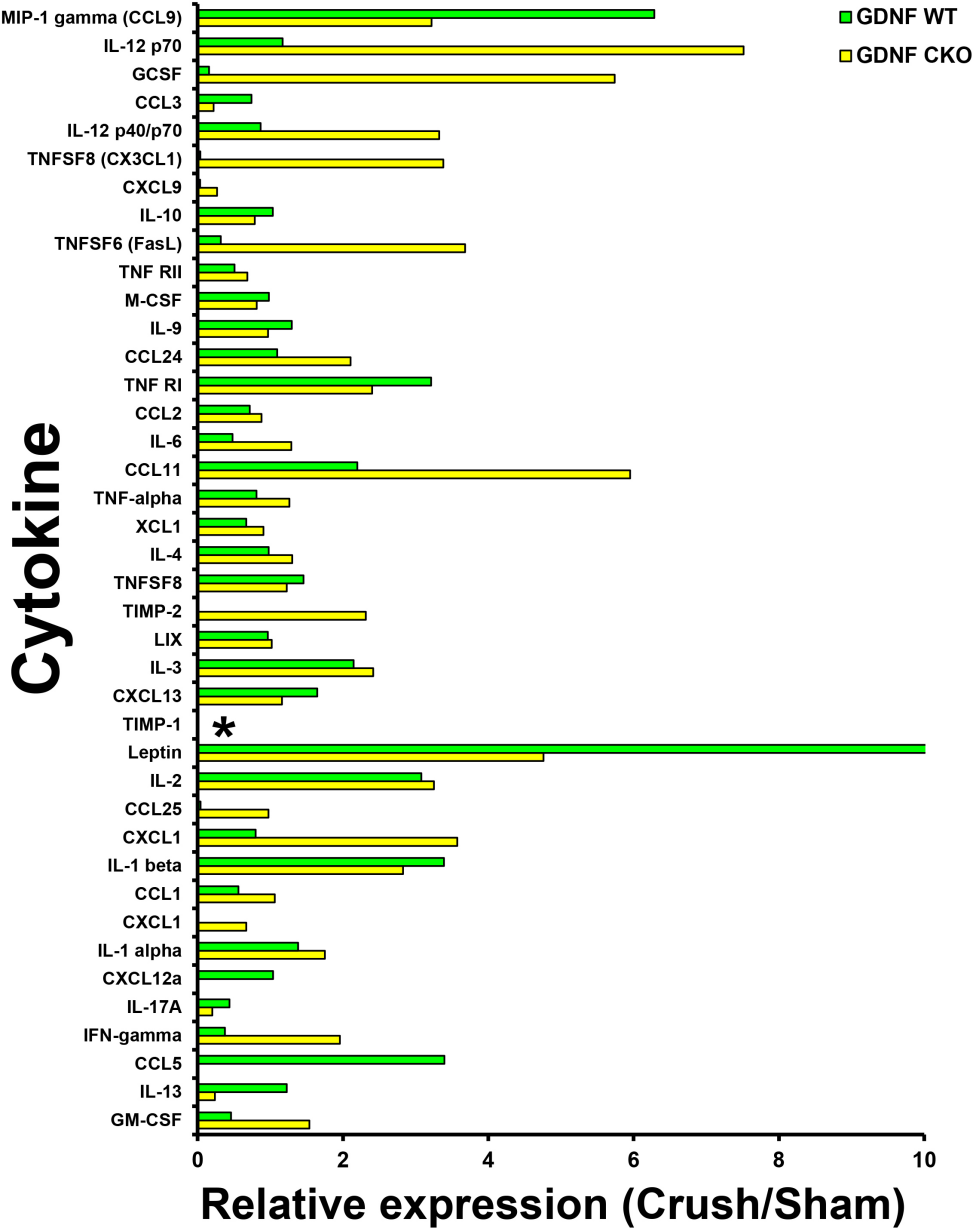
Sciatic nerve cytokine expression profiles in age- and sex-matched *Gdnf* CKO and *Gdnf* WT mice following unilateral crush injury. Bar histograms from a representative whole sciatic nerve homogenate semi-quantitative cytokine array spot densitometry experiment demonstrating relative cytokine expression in injured sciatic nerves compared to uninjured (Sham control) sciatic nerves are shown. TIMP-1 is expressed only in *Gdnf* CKO injured sciatic nerves. * indicates a ratio of infinity. N = 4 (16 sciatic nerves in total).

**Figure 11:**
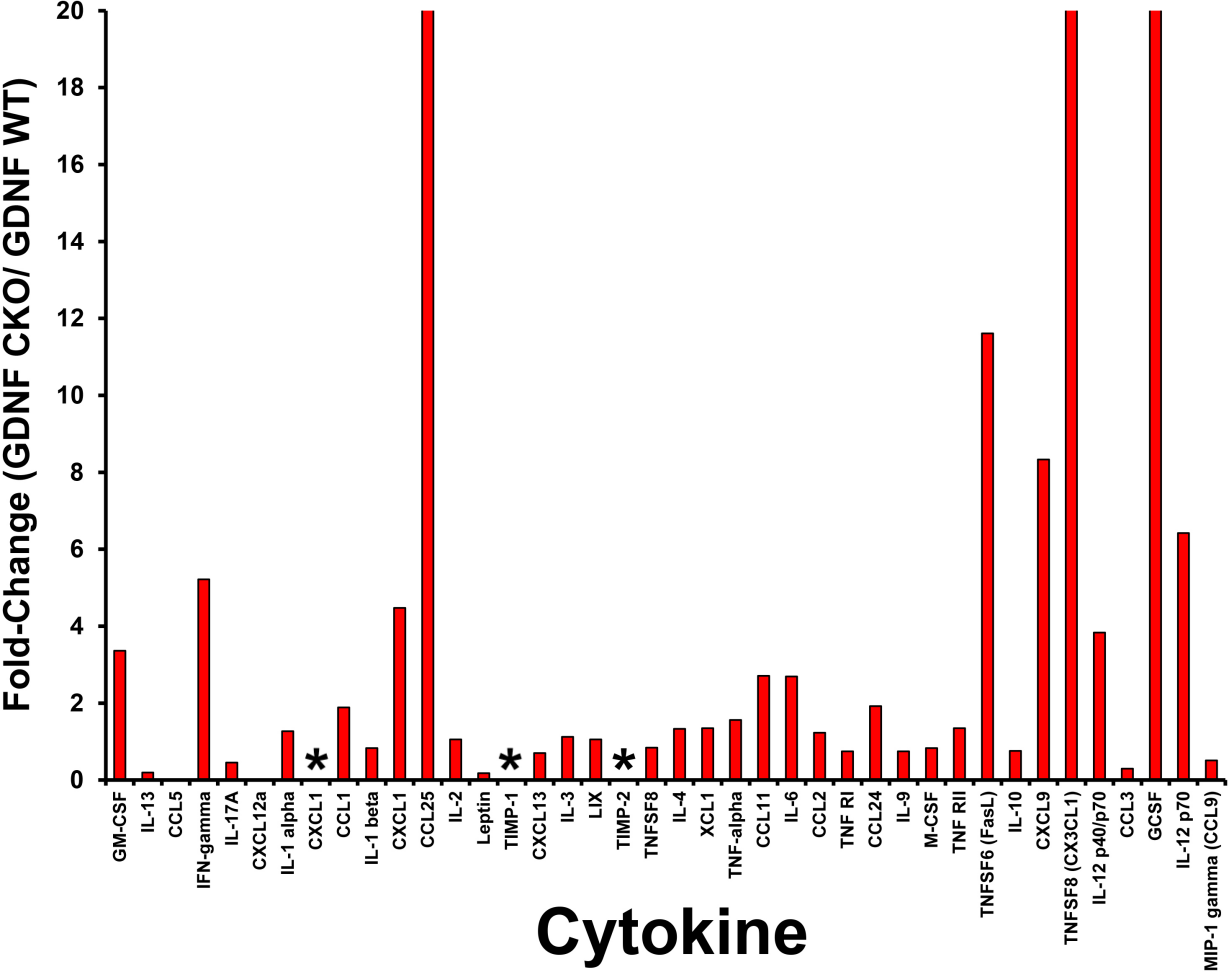
Relative sciatic nerve cytokine expression in *Gdnf* CKO and *Gdnf* WT mice following unilateral crush injury. Bar histograms from a representative whole sciatic nerve homogenate semi-quantitative cytokine array spot densitometry experiment demonstrating relative normalized cytokine expression in injured sciatic nerves from *Gdnf* CKO and *Gdnf* WT mice are shown. High relative cytokine expression in the injured sciatic *Gdnf* CKO mice compared to *Gdnf* WT mice may be associated with subsequent development of chronic nociception. The highest relative expression is seen in CXCL1, TIMP-1 and TIMP-2, with >20-fold increases seen in CCL25, CX3CL1 and GCSF. * indicates a ratio of infinity. N = 4 (16 sciatic nerves).

The unique expression patterns observed from the protein array provide cytokines that we hypothesize are potential crush injury, chronic nociception or axonal recovery biomarkers (raw data, **Figure 9**). Molecules highly expressed in injured *Gdnf* CKO mouse sciatic nerves (i.e. from mice with persistent axonal degeneration, delayed axonal regeneration and chronic nociception) (54, 64) relative to *Gdnf* WT mice, such as CX3CL1 (fractalkine), granulocyte colony stimulating factor (GCSF), tissue necrosis factor superfamily member 6 (TNFSF6, also known as Fas Ligand), interferon-γ, granulocyte-macrophage colony stimulating factor (GM-CSF) and CCL11 could be important pathogenic, predictive or prognostic biomarkers for traumatic neuropathies with chronic pain that may have translational potential to chronic peripheral neuropathies (**Figures 10**, **11**).

Interestingly, we observed that tissue inhibitor of metalloproteinases-1 (TIMP-1) was expressed only in injured *Gdnf* CKO mouse sciatic nerves at day 7 (**Figures 10**, **11**), suggesting it may be an early predictive biomarker of chronic nociception, as suggested in a rat spinal cord injury model (75). Longitudinal measurements of TIMP-1 concentrations in mouse models and patients with correlation using validated nociception measures could determine whether this cytokine is a reliable chronic neuropathic pain biomarker applicable to clinical practice.

## Discussion

Our preliminary data demonstrate that chronic nociception develops in adult Tamoxifen-induced *Gdnf* CKO mice following sciatic nerve crush injury. These mice develop persistent axonal degeneration and blood-nerve barrier permeability, and delayed axonal regeneration due to abrogated Gdnf expression during recovery. SAPP mice develop chronic nociception at expected disease onset, associating nociception with early sciatic nerve leukocyte infiltration and demyelination. Increased sciatic nerve CD11b+ CD45+ leukocyte infiltration occurred in three mouse models of inflammatory and traumatic peripheral neuropathies that recapitulate essential features of human peripheral neuropathies. Interestingly, although increased unilateral lumbar DRG CD11b+ CD45+ leukocyte infiltration was observed in our mouse model of traumatic neuropathy in *Gdnf* CKO mice that develop chronic nociception compared to *Gdnf* WT mice that do not. However, most of the infiltrated CD45+ leukocytes in the DRGs were CD11b-, implying that leukocyte trafficking at the site of peripheral nerve injury may differ significantly from other neural sites that could regulate nociceptive signal processing after injury.

Systemic CD11b antagonism with a function-neutralizing monoclonal antibody resulted in resolution of chronic nociception in our mouse model of traumatic neuropathy induced in adult *Gdnf* CKO mice, and we did not observe drug reward behaviors in healthy adult mice treated with this monoclonal antibody. Coupled with the observation that there were increased endoneurial CD11b+ leukocytes observed in the sural nerves of vasculitic neuropathy, CIDP and HIV neuropathy patients associated with chronic neuropathic pain, targeted therapeutic modulation of CD11b-mediated leukocyte trafficking could effectively treat chronic neuropathic pain associated with specific infectious, inflammatory and traumatic peripheral neuropathies.

However, several limitations exist that affect the translational potential of cytokine, chemokine and integrin expression/signaling from mouse models to humans. Reflexive neurobehavioral nociception does not equate to the complex chronic neuropathic pain experience in patients. Furthermore, in-bred mouse strains do not reflect the biological diversity seen in peripheral neuropathy patients with chronic pain. Nerves biopsies are not routinely performed in chronic neuropathic pain patients, so the full repertoire of biologically relevant cytokine/chemokine signaling and leukocyte infiltration in these disorders has not been elucidated. In order to better understand chronic neuropathic pain using murine models, longitudinal quantitative assessments of sciatic nerve and lumbar DRG cytokine/chemokine expression, relevant signaling pathways and leukocyte infiltration should be studied in correlation to reflexive and non-reflexive (e.g. facial grimacing) (76–78) neurobehavioral nociception outcomes in multiple models in a scientifically rigorous blinded manner. This is a human “clinical trials” approach that would support increased comprehension of the cellular and molecular determinants and signaling pathways important for chronic nociception in inflammatory and traumatic neuropathies over time, and improve translational potential to peripheral neuropathy patients with chronic neuropathic pain.

Biomarkers could be diagnostic, prognostic, predictive, measures of susceptibility or risk, serve as indicators of treatment response, or indicators of adverse responses to treatment. The pathogenic role of CX3CL1 in chronic pain and inflammation is well established (79). TIMP-1 belongs to a family of cytokines that are expressed in response to multiple cytokine release in tissues and are well known for their role in extracellular remodeling by inhibiting matrix metalloproteinases (80). Identifying molecular profiles (preferably in serum, plasma or on circulating leukocytes) at well-defined disease stages in these peripheral neuropathies using transcriptomics (81), proteomics (82), flow cytometry or a combination thereof, supported by bioinformatics analyses to detect pathway similarities and differences, could advance current knowledge. This may lead to more disease-targeted personalized treatments and more precise objective disease measures in chronic neuropathic pain. Such knowledge would be enhanced by spatial transcriptomics of well-characterized peripheral nerve biopsies from chronic neuropathic pain patients at single cell resolution referenced to histologically normal adult peripheral nerve biopsies, using techniques recently published in decedent human DRGs (83).

Studies are needed to better understand clinically relevant cellular and molecular determinants, as well as signaling pathways responsible for chronic neuropathic pain development. These could be generalized or unique to specific peripheral neuropathies or patient subpopulations. Persistent leukocyte infiltration mediated by cytokines, chemokines and leukocyte integrins are potentially relevant to chronic neuropathic pain development and persistence. Rigorous studies guided by observational data from well-characterized human peripheral neuropathies that align animal model investigation with diagnostic and therapeutic clinical trials in human populations should help increase the translational usefulness of these animal models. The quest remains to identify effective targeted treatments with limited addiction potential and clinically relevant and applicable biomarkers for chronic neuropathic pain in peripheral neuropathy patients.

## Data availability statement

The raw data supporting the conclusions of this article will be made available by the authors, without undue reservation.

## Ethics statement

The animal study was reviewed and approved by University of Alabama at Birmingham Institutional Animal Care and Use Committee.

## Author contributions

CD and EEU were involved in study conception and experimental design, as well as data acquisition, analysis and interpretation, and in generating figures and drafting the manuscript. All authors contributed to the article and approved the submitted version.

## Funding

Data shown in this manuscript were supported by projects funded by the United States of America National Institutes of Health (NIH) grants R21 NS073702 [2011-2014], R21 NS078226 [2012-2015], R01 NS075212 [2012-2017] and institutional funds from the University of Alabama at Birmingham. The funding sources had no involvement in the conduct of the research, manuscript preparation, data collection/analyses or decision to submit this work for publication. The content is solely the responsibility of the authors and does not necessarily represent the official views of the NIH.

## Acknowledgments

Special thanks to current and past members and scientific collaborators of the Neuromuscular Immunopathology Research Laboratory (NIRL) at Baylor College of Medicine and the University of Alabama at Birmingham (UAB) for insightful discussions and experimental work with the murine models of inflammatory and traumatic peripheral neuropathies, including reflexive neurobehavioral nociception and conditioned place preference testing. Special thanks to past and current employees of the Shin J Oh Muscle and Nerve Histopathology Laboratory, UAB for initially processing archived cryopreserved human sural nerves and histopathology slides.

## Conflict of interest

EEU has a non-exclusive commercial license (held by Baylor Licensing Group) for simian virus-40 large T-antigen immortalized human endoneurial endothelial cells and has received royalties from Springer Science + Business Media for an edited book on laboratory protocols that describes a flow-dependent *in vitro* human blood-nerve barrier leukocyte trafficking assay. These financial interests do not constitute direct competing interests with this work.

The remaining author declares that the research was conducted in the absence of any commercial or financial relationships that could be construed as a potential conflict of interest.

## Publisher’s note

All claims expressed in this article are solely those of the authors and do not necessarily represent those of their affiliated organizations, or those of the publisher, the editors and the reviewers. Any product that may be evaluated in this article, or claim that may be made by its manufacturer, is not guaranteed or endorsed by the publisher.
